# Molecular determinants of TRPM8 function: key clues for a cool modulation

**DOI:** 10.3389/fphar.2023.1213337

**Published:** 2023-06-14

**Authors:** María Pertusa, Jocelyn Solorza, Rodolfo Madrid

**Affiliations:** ^1^ Departamento de Biología, Facultad de Química y Biología, Universidad de Santiago de Chile, Santiago, Chile; ^2^ Millennium Nucleus of Ion Channel-Associated Diseases (MiNICAD), Santiago, Chile; ^3^ Millennium Nucleus for the Study of Pain (MiNuSPain), Santiago, Chile; ^4^ Centro de Bioinformática, Simulación y Modelado (CBSM), Facultad de Ingeniería, Universidad de Talca, Talca, Chile

**Keywords:** cold, menthol, icilin, WS-12, cryo-EM structures, ion channel

## Abstract

Cold thermoreceptor neurons detect temperature drops with highly sensitive molecular machinery concentrated in their peripheral free nerve endings. The main molecular entity responsible for cold transduction in these neurons is the thermo-TRP channel TRPM8. Cold, cooling compounds such as menthol, voltage, and osmolality rises activate this polymodal ion channel. Dysregulation of TRPM8 activity underlies several physiopathological conditions, including painful cold hypersensitivity in response to axonal damage, migraine, dry-eye disease, overactive bladder, and several forms of cancer. Although TRPM8 could be an attractive target for treating these highly prevalent diseases, there is still a need for potent and specific modulators potentially suitable for future clinical trials. This goal requires a complete understanding of the molecular determinants underlying TRPM8 activation by chemical and physical agonists, inhibition by antagonists, and the modulatory mechanisms behind its function to guide future and more successful treatment strategies. This review recapitulates information obtained from different mutagenesis approaches that have allowed the identification of specific amino acids in the cavity comprised of the S1-S4 and TRP domains that determine modulation by chemical ligands. In addition, we summarize different studies revealing specific regions within the N- and C-terminus and the transmembrane domain that contribute to cold-dependent TRPM8 gating. We also highlight the latest milestone in the field: cryo-electron microscopy structures of TRPM8, which have provided a better comprehension of the 21 years of extensive research in this ion channel, shedding light on the molecular bases underlying its modulation, and promoting the future rational design of novel drugs to selectively regulate abnormal TRPM8 activity under pathophysiological conditions.

## 1 Introduction

Until 2002, little was known about how temperature drops activate the sub-population of somatosensory fibers responsible for cold detection. That year, Nobel Laureates David Julius and Ardem Patapoutian published two seminal studies with their independent findings, describing the molecular machine that allows mammals to detect cold (McKemy et al., 2002; Peier et al., 2002). Using two different strategies, they found the answer in the TRP channel TRPM8, the eighth member of the Transient Receptor Potential Melastatin family, providing a new and exciting candidate to help understand the molecular logic of cold sensing. They also showed that this Ca^2+^-permeable non-selective cation channel, expressed in trigeminal ganglia (TG) and dorsal root ganglia (DRG) neurons, was activated by natural and artificial cooling compounds (McKemy et al., 2002; Peier et al., 2002), explaining Hensel and Zotterman’s foundational observations that menthol sensitizes and potentiates the cold-evoked electrical responses of cold thermoreceptor fibers (Hensel and Zotterman, 1951). Shortly after, in 2004, two independent groups reported that TRPM8 is also a voltage-dependent channel activated by membrane depolarization (Brauchi et al., 2004; Voets et al., 2004). These studies revealed that TRPM8 activation by cold and menthol promotes a shift in its activation curve towards negative membrane potentials, increasing the open probability at physiologically relevant membrane potentials (Brauchi et al., 2004; Voets et al., 2004). However, evidence demonstrating TRPM8 activation by cold and its expression in cold thermoreceptor neurons was insufficient to unequivocally establish its contribution to cold sensing in mammals. The generation of three different TRPM8 knockout mice (TRPM8^−/−^) revealed that animals lacking functional expression of the TRPM8 channel display an evident impairment in their ability to avoid cold temperatures in a temperature preference chamber and an attenuated response to evaporative cooling, highlighting its relevance as a crucial molecular cold transducer (Bautista et al., 2007; Colburn et al., 2007; Dhaka et al., 2007).

In addition to its role in innocuous cold transduction, selective ablation of TRPM8-expressing neurons also yielded animals with a marked reduction in cold sensitivity at the noxious range of low temperatures, supporting the idea that TRPM8 has an important role in cold-induced pain (Knowlton et al., 2013; Pogorzala et al., 2013). Interestingly, cold or topical menthol are commonly used for pain relief, suggesting that the TRPM8 channel is also involved in this analgesic effect (Proudfoot et al., 2006; Liu et al., 2013). Therefore, depending on the subpopulation of neurons where TRPM8 is expressed and the neural pathway involved, this channel emerges as a critical molecular component in innocuous cool sensation, cold nociception, and cold-induced analgesia. A study categorizing mouse primary sensory neurons through single-cell RNA sequencing found three different subtypes of neurons expressing TRPM8 channels (Zeisel et al., 2018). Whether these subtypes are behind the different functions of the TRPM8 expressing fibers still needs to be further clarified (Kupari and Ernfors, 2023). Moreover, the development and maintenance of painful cold hypersensitivity in response to axonal damage have been linked to increased TRPM8 expression (Xing et al., 2007; Su et al., 2011; Piña et al., 2019), and TRPM8^−/−^ animals display reduced nocifensive behavior in response to nerve injury (Bautista et al., 2007; Colburn et al., 2007; Knowlton et al., 2010). Additionally, polymorphisms in the TRPM8 gene have been related to migraine by genome-wide association studies (Freilinger et al., 2012; Ling et al., 2019). In line with this observation, TRPM8 activation in the *dura mater* produced migraine-like behavior in rats, which was sensitive to drugs used to treat this pathology in humans (Burgos-Vega et al., 2016). Interestingly, it has been shown that TRPM8 has a protective role in males in a mouse model of migraine (Alarcón-Alarcón et al., 2022). In the cornea, TRPM8-expressing neurons act not only as cold-sensing neurons but also as humidity detectors and osmosensors of the eye’s surface, where TRPM8 activity accounts for the ongoing firing that stimulates basal tearing secretion and modulates the regular blinking rate (Parra et al., 2010; Quallo et al., 2015). These relevant roles of TRPM8-expressing thermoreceptors in corneal physiology can become powerful targets to treat tear film-associated pathologies (Belmonte et al., 2017).

TRPM8 channels are also involved in maintaining core body temperature (Tc). Topical menthol application induces thermogenic responses: shivering-like muscle activity, increased oxygen consumption, tail-skin vasoconstriction, and heat-seeking behavior (Tajino et al., 2007). In contrast, the inhibition of TRPM8 channels reduces Tc (Camila Almeida et al., 2012; Gavva et al., 2012). However, TRPM8-deficient mice show only mild impairment in their ability to maintain Tc, indicating that the trigger of heat-generating thermoregulatory effectors as a response to environmental cold also involves TRPM8-independent mechanisms (Reimúndez et al., 2018). In the same study, the authors reported that TRPM8^−/−^ mice housed at 21°C experienced late-onset obesity, probably due to diurnal hyperphagia and reduction of fat oxidation, suggesting that TRPM8 could regulate an optimal ingestive thermoregulatory response (Reimúndez et al., 2018). In addition to the physiological and physiopathological roles mentioned above, TRPM8 has also emerged as a crucial player in other pathologies. For instance, an overactive bladder aggravated by cold temperatures results from increased expression of TRPM8 channels on bladder afferent nerve fibers, highlighting the essential role of TRPM8 in the lower urinary tract (Mukerji et al., 2006). Interestingly, different studies reported an abnormal TRPM8 function in several forms of cancer, including prostate, pancreatic, breast, lung, and colon cancer (reviewed by (Ochoa et al., 2023)); however, in some cases, the contribution to the pathology is still not entirely understood.

The increasing evidence of TRPM8 participation in pathological contexts has made this ion channel an attractive molecular target to treat highly prevalent diseases. However, specific and potent TRPM8 chemical modulators for clinical use are lacking (González-Muñiz et al., 2019; Izquierdo et al., 2021). This review focuses on recapitulating the current information on the molecular determinants underlying the modulation of TRPM8 activity obtained from several mutagenesis strategies, chimeric proteins, and the recent cryo-Electron Microscope (cryo-EM) structures, that should be considered to shed light for the future therapeutic developments entailing this polymodal ion channel.

## 2 TRPM8 structure

**FIGURE 1:**
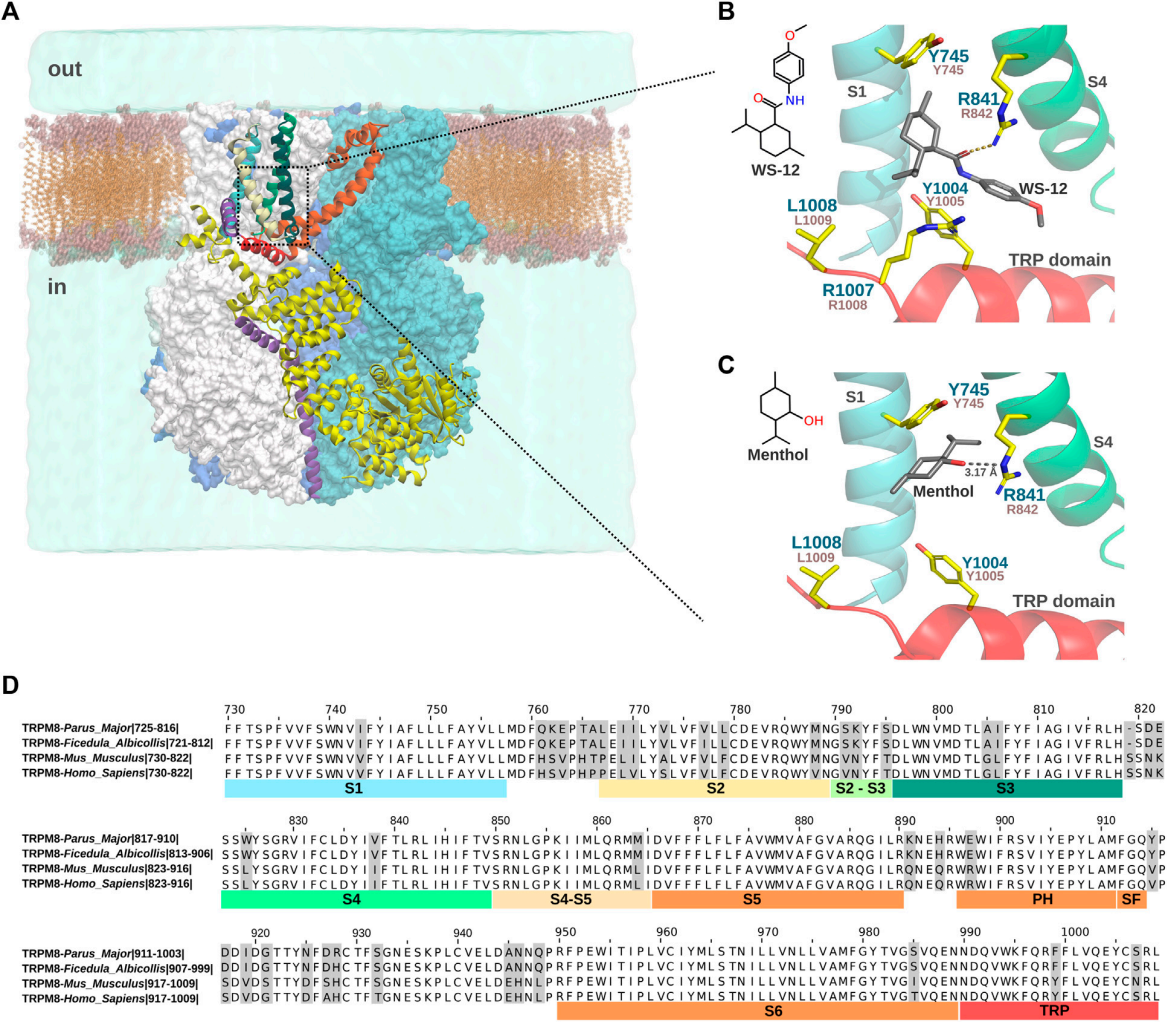
Menthol and WS-12 binding site in the VSLD of FaTRPM8. **(A)**. Representation of the TRPM8 system embedded in the lipid membrane and water. The four subunits are shown in surface representation. One monomer with secondary structure elements is shown for reference (S1 is highlighted in cyan, S2 in white, S3 in light green, S4 in dark green, S5 and S6 in orange, and the TRP domain in red). The dotted box indicates the ligand binding site of TRPM8 formed by the S1-S4 transmembrane segments (VSLD) and the TRP domain. **(B)**. Close-up view of the WS-12 binding in the VSLD cavity (PDB ID: 6NR2). Key residues involved in menthol-/WS-12-dependent TRPM8 activation mentioned in the text and Table 2 are shown. Residue numbers correspond to FaTRPM8 (dark green) and MmTRPM8 (brown). For clarity, the S2 and S3 transmembrane segments were omitted. The side chain of residue L1008 was added using Pymol v2.5.4. **(C)**. General description of the computational modeling of menthol binding by Xu et al., 2020, using a model based on the FaTRPM8 in complex with WS-12 (PDB ID: 6NR2). For clarity, S2 and S3 were omitted, and the side chain of residue L1008 was added using Pymol v2.5.4. **(D)**. Alignment of S1-S6 and TRP domain of *Parus major*, *Ficedula albicollis*, *Mus musculus*, and *Homo sapiens* orthologs of TRPM8 using Jalview (Waterhouse et al., 2009). Numbers correspond to residues in MmTRPM8 channel.

One of the most important milestones in the field has been the determination of TRPM8 structures by cryo-EM (Yin et al., 2018; 2019; 2022; Diver et al., 2019; Zhao et al., 2022) (Table 1). The functional TRPM8 channel requires the assembly of four identical subunits containing cytosolic N-terminal and C-terminal domains and a transmembrane domain with six (S1-S6) helices, which contribute to the tetrameric assembly of the channel protein (Yin et al., 2018; 2019; 2022; Diver et al., 2019; Zhao et al., 2022) (Figure 1A). The N-terminus contains four Melastatin Homology Regions (MHR), named for their sequence similarity exhibited by members of the TRPM family (Fleig and Penner, 2004). Part of the region preceding MHR1, MHR1 itself, and MHR2 form an alpha/beta-fold domain (MHR1/2). In contrast, MHR3 and MHR4 consisted of helix-turn-helix motifs (Yin et al., 2018; Diver et al., 2019; Zhao et al., 2022). Following the last MHR, cryo-EM structures revealed a pre-S1 domain in avian and mammalian TRPM8 channels. This region consists of a cytosolic pre-S1 helix, a helix-turn-helix motif, and a helix connecting to S1, presumably in the membrane region (Yin et al., 2018; Diver et al., 2019; Zhao et al., 2022). Like other thermo-TRP channels such as TRPV1 and TRPV2 (Liao et al., 2013; Zubcevic et al., 2016), the S1 to S4 constitutes a voltage-sensor-like domain (VSLD) (Yin et al., 2018), and the S5–S6 region forms the pore (Yin et al., 2018; Diver et al., 2019; Zhao et al., 2022). It is important to mention that before the cryo-EM structures, the algorithms used to predict the TRPM8 transmembrane domains pinpointed this pre-S1 domain as the S1. For this reason, most studies before 2018 placed tyrosine 745, a residue with an essential role in TRPM8 activation by chemical agonists, within the S2 when this amino acid is located in the S1 (Bandell et al., 2006; Mälkiä et al., 2009; Beccari et al., 2017). Akin to the previously determined TRPV structure, TRPM8 exhibits a domain-swapped arrangement, where the VSLD of one subunit interacts with the pore domain of the neighboring monomer (Yin et al., 2018; Diver et al., 2019; Zhao et al., 2022). The cytosolic C-terminus contains the TRP domain (comprising positions 992–1009), sandwiched between the S4-S5 linker (above) and the MHR4 domain (below) (Yin et al., 2022; Zhao et al., 2022). The C-terminal part of the TRP domain and S1 to S4 integrate the VSLD cavity where agonists and antagonists bind (Yin et al., 2018; Diver et al., 2019; Zhao et al., 2022). Finally, the TRP domain is followed by three further helices (Yin et al., 2018; Diver et al., 2019; Zhao et al., 2022). The latter forms a coiled-coil motif that drives the tetramerization of TRPM8 channels (Tsuruda et al., 2006).

**TABLE 1:** Summary of Cryo-EM TRPM8 structures.

PDB ID	Resolution (Å)	Ligands	Species	References
**6BPQ**	4.1	Free	*Ficedula albicollis*	Yin et al. (2018)
**6NR4**	4.3	Icilin, PI_(4,5)_P_2_, Ca^2+^	*Ficedula albicollis*	Yin et al. (2019)
**6NR2**	4	WS-12, PI_(4,5)_P_2_	*Ficedula albicollis*	Yin et al. (2019)
**6NR3**	3.4	Icilin, PI_(4,5)_P_2_, Ca^2+^	*Ficedula albicollis*	Yin et al. (2019)
**8E4Q**	3.51	PI_(4,5)_P_2_	*Ficedula albicollis*	Yin et al. (2022)
**6O6A**	3.6	Free	*Parus major*	Diver et al. (2019)
**6O77**	3.2	Ca^2+^	*Parus major*	Diver et al. (2019)
**6O6R**	3.2	AMTB	*Parus major*	Diver et al. (2019)
**6O72**	3	TC-I 2014	*Parus major*	Diver et al. (2019)
**7WRA**	2.98	Free	*Mus musculus*	Zhao et al. (2022)
**7WRB**	2.88	Ca^2+^	*Mus musculus*	Zhao et al. (2022)
**7WRC**	3.21	Icilin, PI_(4,5)_P_2_, Ca^2+^	*Mus musculus*	Zhao et al. (2022)
**7WRD**	2.98	Icilin, Ca^2+^	*Mus musculus*	Zhao et al. (2022)
**7WRE**	2.52	Icilin, Ca^2+^	*Mus musculus*	Zhao et al. (2022)
**7WRF**	3.04	Icilin, PI_(4,5)_P_2_, Ca^2+^	*Mus musculus*	Zhao et al. (2022)
**8E4L**	3.32	C3, AITC, PI_(4,5)_P_2_	*Mus musculus*	Yin et al. (2022)
**8E4M**	3.44	C3, PI_(4,5)_P_2_	*Mus musculus*	Yin et al. (2022)
**8E4N**	3.07	PI_(4,5)_P_2_	*Mus musculus*	Yin et al. (2022)
**8E4O**	3.43	PI_(4,5)_P_2_ (putative)	*Mus musculus*	Yin et al. (2022)
**8E4P**	3.59	Free	*Mus musculus*	Yin et al. (2022)

## 3 How TRPM8 is activated by exogenous agonists: Lessons from single point mutagenesis to structural data

### 3.1 Menthol and WS-12

One of the questions extensively explored in the first years after cloning TRPM8 was the molecular determinants responsible for its activation by cold and chemical agonists. The experimental approaches were mainly two: single-point mutagenesis and constructing chimeras. The first one successfully identified residues involved in TRPM8 activation by exogenous or endogenous chemical agonists. In that regard, Bandell and coworkers made a breakthrough when they identified residues involved in activating the channel by menthol. The screening of 14,000 TRPM8 clones obtained by high-throughput mutagenesis revealed that Y745H, Y1005F, and L1009R mutations rendered channels activated by temperature drops and insensitive to menthol (Bandell et al., 2006). To elucidate whether the cause behind the menthol-insensitive phenotype is the loss of tyrosine and leucine side chains or the residues used for the replacement, the authors investigated the impact of different substitutions generating Y745A, Y745F, Y1005F, Y1005A, L1009P, and L1009A TRPM8 mutants (Table 2). While the Y745A mutation also produced a menthol-insensitive channel, introducing a phenylalanine did not ultimately abolish menthol activation when this agonist was used at high concentrations (Bandell et al., 2006). Likewise, the comparison of Y1005F and Y1005A mutants revealed a more pronounced effect in the menthol response of the Y1005A channel. These results suggest that the aromatic ring and the hydroxyl of these tyrosine side chains could contribute to the menthol sensitivity of TRPM8 channels (Bandell et al., 2006). In contrast, L1009P and L1009A behave as wild-type channels, indicating that rather than the leucine *per se*, exchange for an arginine prevents menthol-induced TRPM8 activation (Bandell et al., 2006). Another residue involved in the TRPM8 menthol response is R842 (Voets et al., 2007). The first evidence of this was described in a study exploring the effect of charge-neutralizing mutations in the S4. The authors observed that the R842A mutant exhibited a decrease in menthol sensitivity along with a reduction in the cold-evoked response and the gating charge, indicating that this mutation affected more than just menthol response (Voets et al., 2007). However, a subsequent study reported that substituting the arginine for histidine at the same position (R842H) rendered a mutant channel exhibiting a strongly impaired response to menthol, without apparent changes in voltage dependence and cold responses (Janssens and Voets, 2011). The substitution of the R842 by another positively charged amino acid (R842K) revealed a modest increase in the EC_50_ value for menthol (Voets et al., 2007; Xu et al., 2020).

**TABLE 2:** Mutations of residues involved in TRPM8 activation by menthol without major alterations in their cold-evoked responses.

Mutant	Domain	Menthol activation	Ortholog	References
**Y745A**	S1	No	MmTRPM8	Bandell et al. (2006)
**Y745H**	S1	No	MmTRPM8	Bandell et al. (2006)
				Mälkiä et al. (2009)
				Xu et al. (2020)
**Y745H**	S1	No	HsTRPM8	Babes et al. (2017)
**Y745F**	S1	Reduced	HsTRPM8	Bandell et al. (2006)
**R842A^a^**	S4	Strongly reduced	HsTRPM8	Voets et al. (2007)
**R842H**	S4	Strongly reduced	HsTRPM8	Voets et al. (2007)
				Janssens and Voets (2011)
**R842K**	S4	Slightly reduced	HsTRPM8	Voets et al. (2007)
**R842K**	S4	Slightly reduced	MmTRPM8	Xu et al. (2020)
**Y1005F**	TRP-domain	Reduced	MmTRPM8	Bandell et al. (2006)
**Y1005A**	TRP-domain	No	MmTRPM8	Bandell et al. (2006)
**L1009R**	TRP-domain	Strongly reduced	MmTRPM8	Bandell et al. (2006)
**L1009A**	TRP-domain	Yes	MmTRPM8	Bandell et al. (2006)
**L1009P**	TRP-domain	Yes	MmTRPM8	Bandell et al. (2006)
^**1009**^ **PAA** ^**1011**^	TRP-domain	Slightly reduced	MmTRPM8	Bandell et al. (2006)

^a^ This mutant also showed a reduction in the cold response and the gating charge.

The lack of menthol-dependent TRPM8 activation generated by substituting these residues could result from alterations of the menthol binding site, or because they are responsible for the conformational changes upon binding that participate in the gating steps downstream of this interaction. For instance, the substantial reduction in the menthol response exhibited by L1009R was explained not as changes in the EC_50_ but as a severe decrease in the efficacy (Bandell et al., 2006), which could suggest that rather than affecting the binding, this mutation abrogates the ability of TRPM8 to convey the menthol interaction and result in channel opening. That idea was corroborated by displacement studies using tritiated menthol, which showed that the Y745H mutant abolished specific menthol binding exhibited by the wild-type channel but not the L1009R mutant (Voets et al., 2007). However, a different study where the direct binding of menthol of the purified HsTRPM8 VSLD domain was assessed by nuclear magnetic resonance spectroscopy, far-UV circular dichroism, and microscale thermophoresis revealed that Y745H and R842H mutants retain their ability to bind menthol. This result would suggest that Y745 and R842 are not essential for binding but rather for the structural coupling that leads to TRPM8 gating (Rath et al., 2016). Therefore, additional structural data are required to draw conclusions regarding the characterization of the agonist binding site. Even though we still lack a menthol-bound TRPM8 structure channel, the recent high-resolution cryo-EM structures of an avian TRPM8 ortholog from *Ficedula albicollis* (FaTRPM8) revealed that a menthol analog, WS-12, with higher efficacy, potency, and selectivity than menthol (Bödding et al., 2007), binds to a cavity formed by transmembrane domains S1-S4 and the TRP domain wedged between Y745 and Y1004 (Yin et al., 2019). Specifically, in the presence of phosphatidylinositol-4,5-biphosphate (PI(_4,5_)P_2_), side chains of R841, Y1004, and R1007 (R842, Y1005, and R1008 in mouse and human orthologs) interact with the WS-12 molecule (Figure 1B). Regarding menthol binding, the computational docking of this agonist to the WS-12-PI(_4,5_)P_2_ bound TRPM8 structure (PDB ID:6NR2, Table 1), revealed a predicted hydrogen bond between the hydroxyl group of menthol and the R842 side chain. This approach also showed different van der Waals interactions with several residues of the ligand pocket, Y745 among them, corroborating the relevance of Y745 and R842 in this binding (Xu et al., 2020) (Figure 1C). In this study, the authors also proposed that menthol disrupted the interactions established between Y745, R842, and D802 residues in the absence of a chemical ligand, triggering the conformational changes that lead to TRPM8 activation (Xu et al., 2020). However, regarding the role of D802, located in the S3, in TRPM8 activation by menthol, it has to be noted that mutations of D802 did not alter TRPM8 menthol-evoked responses (Chuang et al., 2004; Kühn et al., 2013).

### 3.2 Icilin

Along with menthol, icilin is one of the most used chemical agonists in TRPM8 research. In contrast to menthol, full TRPM8 activation induced by icilin requires intracellular free Ca^2+^ (McKemy et al., 2002; Chuang et al., 2004; Kühn et al., 2009; Zakharian et al., 2010). To find the residues involved in icilin-induced TRPM8 activation, studies took advantage of the lack of icilin responses exhibited by the chicken ortholog (*Gallus gallus* TRPM8, GgTRPM8), identifying G805 within the S3 as the residue that bestows icilin-sensitivity to mammalian TRPM8 channels (Chuang et al., 2004). Additionally, the mutations of N799 and D802 by alanine yielded channels still responsive to cold, menthol, and Cooling Agent-10 but insensitive to icilin (Chuang et al., 2004; Beccari et al., 2017) (Table 3). Interestingly, menthol-insensitive Y745H and Y745A mutations also abrogate icilin response, indicating that this residue is also relevant to TRPM8 activation by this compound (Bandell et al., 2006; Beccari et al., 2017). These findings were supported by the icilin-PI(_4,5_)P_2_-Ca^2+^ complex obtained by Yin and coworkers (PDB ID.6NR4, Table 1), where it is shown that this agonist was surrounded by Y745 in the S1 and Y1005 within the TRP domain (Yin et al., 2019). In addition, similarly to WS-12 and menthol, icilin interacts with R841 (R842 in the mouse ortholog, *Mus musculus* TRPM8, MmTRPM8) through a hydrogen bond (Yin et al., 2019; Zhao et al., 2022). Corroborating the relevance of this specific interaction, R842Q, R842K and R842N mutants did not respond to icilin (Zhao et al., 2022), suggesting that despite the differences between these agonist structures, some molecular mechanisms for activation by chemical agonists are shared. The icilin-PI(_4,5_)P_2_-Ca^2+^ structure also showed the interaction of this agonist with H844 (H845 in MmTRPM8) (Yin et al., 2019). In agreement with this observation, H844A mutation reduced icilin-evoked currents without apparent alterations in activation by WS-12 (Yin et al., 2019), corroborating its specific role in icilin responses. It has been proposed that the C-terminal part of S4 adopts a 3_10_ helical conformation which facilitates the interaction of R842 and H845 to icilin (Yin et al., 2019; 2022; Zhao et al., 2022).

**TABLE 3:** Mutations of residues involved specifically in TRPM8 activation by icilin.

Mutant	Domain	Icilin activation	Ca^2+^ coordination	Ortholog	References
**Y745A^a^**	S1	No	No	HsTRPM8	Beccari et al. (2017)
**Y745H^a^**	S1	No	No	MmTRPM8	Bandell et al. (2006)
**E773A (E782A)**	S2	No	Yes	PmTRPM8	Diver et al. (2019)
**E782H, E782M, E782R**	S2	No	Yes	MmTRPM8	Zhao et al. (2022)
**Q776A (Q785A)**	S2	No	Yes	PmTRPM8	Diver et al. (2019)
**Y784A (Y793A)**	S2-S3 linker	No	Yes	PmTRPM8	Diver et al. (2019)
**Q785H, Q785L, Q785M, Q785N, Q785R**	S2	No	Yes	MmTRPM8	Zhao et al. (2022)
**Q785K, Q785Y**	S2	Reduced	Yes	MmTRPM8	Zhao et al. (2022)
**N790A (N799A)**	S3	No	Yes	PmTRPM8	Diver et al. (2019)
**D796R**	S3	No	No	HsTRPM8	Kühn et al. (2013)
**N799A^b^**	S3	No	Yes	RnTRPM8	Chuang et al. (2004)
**N799A**	S3	No	Yes	HsTRPM8	Beccari et al. (2017)
**N799D^c^**	S3	No	Yes	HsTRPM8	Winking et al. (2012)
**N799D, N799Q**	S3	Yes	Yes	RnTRPM8	Chuang et al. (2004)
**N799E, N799Y**	S3	No	Yes	RnTRPM8	Chuang et al. (2004)
**N799K**	S3	No	Yes	MmTRPM8	Zhao et al. (2022)
**N799H, N799I**	S3	Strongly reduced	Yes	MmTRPM8	Zhao et al. (2022)
**N799L, N799R**	S3	Reduced	Yes	MmTRPM8	Zhao et al. (2022)
**D793A (D802A)**	S3	No	Yes	PmTRPM8	Diver et al. (2019)
**D802A, D802E, D802H, D802K, D802N, D802Q, D802S, D802Y**	S3	No	Yes	RnTRPM8	Chuang et al. (2004)
**D802A**	S3	No	Yes	HsTRPM8	Beccari et al. (2017)
**D802E, D802I, D802L, D802M, D802N, D802Q, D802R, D802S**	S3	No	Yes	MmTRPM8	Zhao et al. (2022)
**D802A**	S3	Strongly reduced	Yes	MmTRPM8	Zhao et al. (2022)
**D802K**	S3	Reduced	Yes	MmTRPM8	Zhao et al. (2022)
**D802N^d^**	S3	No	Yes	HsTRPM8	Winking et al. (2012)
**D802R^d^**	S3	No	Yes	HsTRPM8	Kühn et al. (2013)
**G805A**	S3	No	No	RnTRPM8	Chuang et al. (2004)
^**803**^ **VGAILL** ^**808**^	S3	No	No	HsTRPM8	Kühn et al. (2009)
**F839Y**	S4	Reduced	No	MmTRPM8	Zhao et al. (2022)
**F839R**	S4	No	No	MmTRPM8	Zhao et al. (2022)
**R842K, R842N, R842Q**	S4	No	No	MmTRPM8	Zhao et al. (2022)
**H844A (H845A)**	S4	Reduced	No	FaTRPM8	Yin et al. (2019)
**F839R + H845R**	S4	No	No	HsTRPM8	Kühn et al. (2013)
**Y1005F**	TRP-domain	Yes	No	MmTRPM8	Zhao et al. (2022)
**L1009R^a^**	TRP-domain	No	No	MmTRPM8	Bandell et al. (2006)
^**1009**^ **PAA** ^**1011**^ **^b^**	TRP-domain	Reduced	No	MmTRPM8	Bandell et al. (2006)

In cases where the amino acid numeration differs among species, the position in the human ortholog is indicated in parenthesis. The Ca^2+^ coordination column indicates if the position in the wild-type channel is involved in the coordination of the Ca^2+^ ion.

The studies that characterized these mutants, also reported that:

^a^ This mutation also abrogates TRPM8 responses to other chemical agonists.

^b^ This mutant showed a potentiation of the cold response in presence of icilin.

^c^ This mutant also showed a reduction in the cold and menthol responses.

^d^ This mutant displayed a decrease in its cold-evoked responses.

**FIGURE 2:**
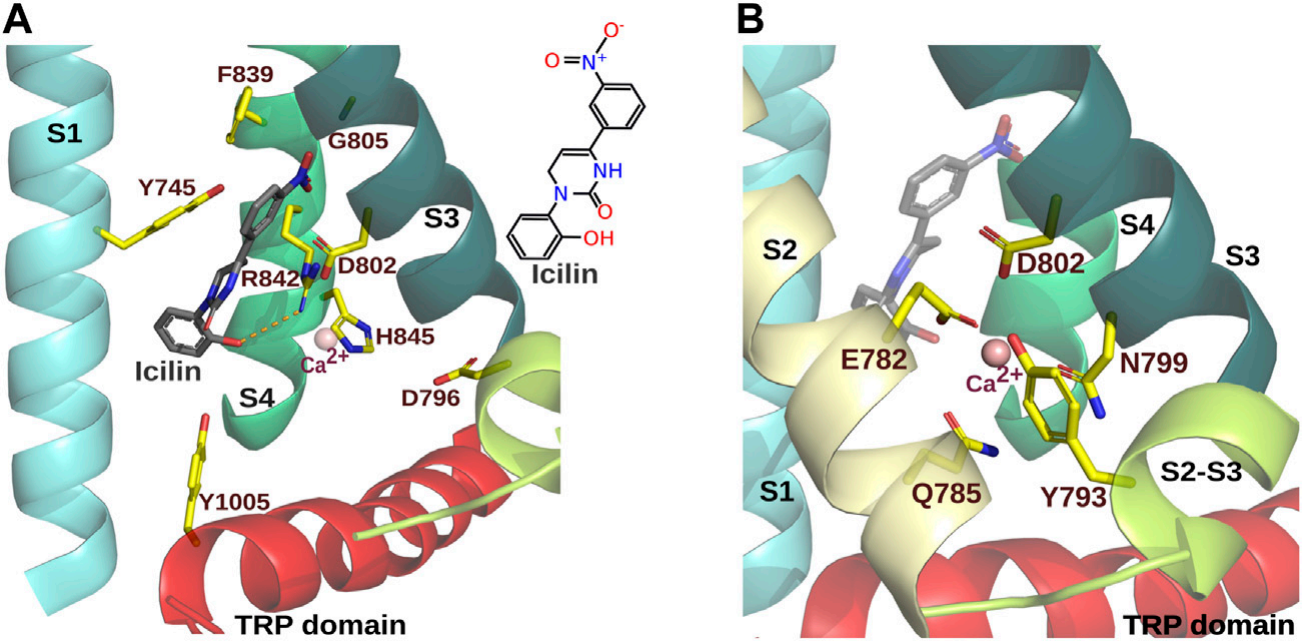
Icilin and Ca^2+^ binding sites in the VSLD of MmTRPM8. **(A).** Icilin binding site of MmTRPM8 (PDB ID: 7WRE). Relevant residues to icilin-evoked TRPM8 responses are indicated (Table 3). The S1 transmembrane segment is highlighted in cyan, S3 in light green, S4 in dark green, and the TRP domain in red. For clarity the S2 was omitted. **(B).** Ca^2+^ ion binding site. Ca^2+^ (depicted as a pink sphere) is coordinated by side chains E782, Q785, N799, and D802 from S2 and S3. In addition, side chain Y793 within the S2-S3 linker is represented (PDB ID: 7WRE) (Visualization in Pymol v2.5.4).

Regarding the role of G805 in the icilin response, it could provide the flexibility required for the S3 rotation necessary to generate the Ca^2+^ binding site, and enlarge the ligand cavity to allow icilin to fit (Yin et al., 2019). The icilin-PI(_4,5_)P_2_-Ca^2+^ structure also indicated that Y1005 hydroxyl forms a hydrogen bond with this compound that could be relevant to the interaction of this agonist (Yin et al., 2019). However, when testing its contribution by replacing this amino acid with phenylalanine in the mouse ortholog, this mutant displayed a similar EC_50_ to the wild type, indicating that at least in the MmTRPM8 channel, this hydroxyl does not participate in icilin binding (Zhao et al., 2022). Moreover, interactions of icilin with the F839 and D802 side chains were also described in the MmTRPM8-Ca^2+^-icilin cryo-EM structure (Zhao et al., 2022) (Figure 2A). Further proving the specific involvement of F839 and D802 in icilin-induced activation, single-point mutagenesis of these residues revealed that this manipulation abolished icilin- but not menthol-induced activation (Zhao et al., 2022). The role of F839 and H845 in icilin-dependent activation was anticipated in a previous study aimed to modify TRPM8 voltage-sensitivity, where a double mutant F839R + H845R was tested. Although menthol elicited wild-type responses in this mutant, a lack of activation by icilin was observed, suggesting the involvement of these residues in the icilin-induced activation (Kühn et al., 2013).

Interestingly, the N799A mutant exhibited potentiation of the cold-evoked response in the presence of icilin, in contrast to mutants D802A or G805A, suggesting a role of N799 in the Ca^2+^-dependent TRPM8 activation by icilin (Chuang et al., 2004). Cryo-EM structures helped to corroborate this hypothesis by identifying positions contributing to the Ca^2+^ coordination site (Diver et al., 2019; Yin et al., 2019; Zhao et al., 2022) (Figure 2B). As expected, mutations of these residues, including N799 and D802, resulted on most occasions in icilin-insensitive channels (Chuang et al., 2004; Winking et al., 2012; Kühn et al., 2013; Diver et al., 2019; Zhao et al., 2022). However, mutants Q785K, Q785Y, N799R, N799L, and D802K, where the icilin response was reduced but not completely abolished, lacked the Ca^2+^-dependent potentiation of icilin-evoked currents exhibited by wild-type MmTRPM8 channels. These findings support the involvement of the Ca^2+^-interacting residues in the icilin response (Zhao et al., 2022). In contrast to N799A, substitution of D802 by alanine abolishes icilin-dependent cold potentiation (Chuang et al., 2004). This difference could be explained because, besides to its role in Ca^2+^ coordination, the D802 side chain showed an anion-π interaction with this agonist (Zhao et al., 2022). Since there is no direct interaction between the Ca^2+^ and icilin, it has been suggested that Ca^2+^ coordination by these residues triggers a conformational arrangement that allows this agonist to bind in the VLSD cavity (Yin et al., 2019; Zhao et al., 2022).

## 4 Molecular determinants of the cold response and temperature-dependent gating: Insights from TRPM8 evolution and mutagenesis analysis to identify functionally relevant regions

One fundamental issue when studying TRPM8 channels is the structural basis of its temperature-dependent gating. Conversely to the activation of TRPM8 by chemical compounds, a single-point mutation appears insufficient to completely abrogate the TRPM8 cold response. In the seminal study of Bandell and coworkers that identified amino acids involved in TRPM8 activation by menthol, the authors also described that some clones displayed a reduced cold response. However, when tested in more detail, these mutations proved to affect sensitivity to cold and menthol (Bandell et al., 2006). Considering this thorough study, where almost all TRPM8 residues were mutated several times, the fact that a cold-insensitive/menthol-responsive phenotype was not observed would suggest that it could be difficult to fully abolish TRPM8 cold-evoked responses from replacing a single amino acid. Building chimeras using cold-insensitive TRPM members could offer an alternative strategy for identifying TRPM8 regions involved exclusively in the cold response. However, this approach assumes that the amino acids mediating the response to cold are not scattered across the whole protein and is not always effective, since replacing even short TRPM8 sequences (20 amino acids long) with other TRPM sequences often yields non-functional channels (Bandell et al., 2006; Voets et al., 2007; Pertusa et al., 2014).

This last obstacle has been overcome by using TRPM8 orthologs. Species-specific differences have generated a wide range of TRPM8 cold-evoked responses across species. Still, their high sequence conservation allows the generation of functional chimeras, where it is possible to identify residues or regions behind the disparities in their thermosensitivity. Although a young *trpm8* gene has been reported in the lungfish *Protopterus annectens*, functional TRPM8 channels have been found in tetrapods but not in bony fishes or invertebrates (Saito and Shingai, 2006; Lu et al., 2022). Except for TRPM8 channels from *Rhinatrema bivittatum* (RbTRPM8), a limbless amphibian, and *Chelonia mydas* (CmTRPM8), a marine turtle that lacked activation upon cooling, the remaining characterized TRPM8 channels from different species retain cold sensitivity despite their differences in their temperature-dependent activation, being mammalian TRPM8 orthologs more robustly activated by the temperature stimulus (Pertusa et al., 2018; Yang et al., 2020; Lu et al., 2022). Therefore, taking advantage of the non-conserved regions of TRPM8 orthologs has been a successful tool for identifying molecular determinants modulating the activation of TRPM8 by cooling.

As a temperature-sensitive ion channel, TRPM8 channel gating is strongly temperature-dependent, exhibiting a 10-degree temperature coefficient (Q_10_)>20 in cellular systems and in lipid bilayers (Brauchi et al., 2004; Zakharian et al., 2010). This parameter is usually used to assess possible variations in the thermosensitivity of TRPM8 mutants. However, since the amplitude of the cold response and the cell temperature threshold are physiologically relevant, mutations that affect these parameters must also be considered. To avoid confusion, it is worth remembering that the temperature threshold is not a temperature value that must be overcome for TRPM8 to open. Instead, it is an operational parameter corresponding to the temperature at which a significant increase in the current, firing rate, or fluorescence of a given cell or sensory neuron is observed (Madrid et al., 2006; Mälkiä et al., 2007; Voets et al., 2007). This section will focus on regions and amino acids involved in TRPM8 cold-evoked response. Interestingly, most of these studies reported alterations in TRPM8 cold activation without significant changes in its gating by other agonists (Brauchi et al., 2006; Voets et al., 2007; Matos-Cruz et al., 2017; Pertusa et al., 2018; Yang et al., 2020; Lu et al., 2022).

Regions or amino acids linked to cold-induced TRPM8 response are found within the N- and C-terminus and the transmembrane domain, suggesting that TRPM8 activation by temperature drops requires concerted structural rearrangements, probably entailing different subunit domains. The C-terminal domain was the first region pinpointed for contributing to TRPM8 thermal activation. Brauchi and others exchanged the C-terminus of the rat TRPM8 channel by the homolog sequence of rat TRPV1, obtaining a construct that, despite exhibiting the same sensitivity to menthol as the wild-type channel, shows more activity at 35°C than at 15°C (Brauchi et al., 2006). The characterization of this chimera revealed a 75 mV shift in the V_1/2_ to more positive potentials when comparing the voltage activation curve obtained at 35°C to the 15°C, in contrast with the left-ward shift displayed by wild-type channels (Brauchi et al., 2006). The inverse temperature phenotype of this chimera suggests that residues within the C-terminal domain define the directionality of the temperature change that allows TRPM8 to open. More recently, Díaz-Franulic and coworkers showed evidence that a cold-induced folding of this domain is required for the temperature-driven gating of the channel (Díaz-Franulic et al., 2020), supporting the idea that the C-terminus plays a key role in the cold sensitivity of TRPM8.

Other studies using orthologs with different cold sensitivities identified the TRPM8 transmembrane domain as an important component for its cold activation. The thirteen-lined ground squirrel is a mammalian hibernator presenting a version of the TRPM8 channel that exhibits a similar activation in response to chemical agonists as other murine TRPM8 channels, but smaller cold-evoked responses (Matos-Cruz et al., 2017). Analysis of chimeras built using rat and squirrel TRPM8 sequences revealed that replacing specific residues scattered with the transmembrane domain of the squirrel channel with the homolog ones from the rat sequence rendered TRPM8 channels activated by temperature similarly to the rat ortholog, indicating the involvement of these amino acids in the cold-evoked responses (Matos-Cruz et al., 2017). Other mutations in the transmembrane domain also impacted TRPM8 activation by cold. Specifically, H845A, R851Q, and R862A mutants shifted their V_1/2_ in cold conditions to more positive membrane potentials without alterations in menthol sensitivity (Voets et al., 2007).

Also based on the construction of functional chimeras, another study took advantage of the complementary functional behavior of MmTRPM8 and GgTRPM8: the mouse ortholog displays larger responses to cold than chicken TRPM8, but the latter shows a higher sensitivity to menthol. It was found that the distinctive cold response of these orthologs is due to non-conserved residues located within the N-terminal part of the pore loop (Pertusa et al., 2018). This observation was also corroborated by comparing TRPM8 from another mammal, the elephant *Loxodonta africana* (LaTRPM8), and the channel from the emperor penguin, *Aptenodytes forsteri* (AfTRPM8). The authors of this study discovered that substituting the V919 (V915 in MmTRPM8), located in the N-terminal portion of the pore loop of mammalian orthologs (Figure 1D), for tyrosine, the corresponding residue in the AfTRPM8 sequence, significantly reduced the maximum cold-evoked current in the elephant TRPM8 channel (Yang et al., 2020). Interestingly, this tyrosine is well conserved in avian species, including chicken, suggesting that the strengthening of TRPM8 cold responsiveness in mammals is linked to changes in the pore loop.

Recently, the cold-insensitiveness of TRPM8 from sea turtle ortholog (CmTRPM8) was critical for determining the relevance of the N-terminus in TRPM8 cold sensitivity. Swapping the first 500 residues from the N-terminal domain of the CmTRPM8 (i.e., MHR from 1 to 3) with the corresponding part of the *Xenopus tropicalis* channel (XtTRPM8), a TRPM8 ortholog that, albeit displaying smaller cold-evoked responses than mammalian TRPM8 channels (Myers et al., 2009; Lu et al., 2022), confers its temperature sensitivity to the resulting construct (MHR1-3XtTRPM8-CmTRPM8) (Lu et al., 2022). Notably, only exchanging the MHR1/2 or the MHR3 failed to bestow cold responsiveness to the resultant chimera, supporting the idea that several residues are involved in TRPM8 activation by cold. MHR1-3XtTRPM8-CmTRPM8 chimera also contains a tyrosine in position 906 in the pore loop sequence, the homologous site of residue 915 in the AfTRPM8, which is responsible for the reduced cold-evoked response compared to mammalian TRPM8 channels. Substituting Y906 with a more hydrophobic amino acid in this chimera generated TRPM8 channels showing enhanced cold-evoked responses, similar to those observed in the penguin TRPM8 ortholog when Y915V mutation is introduced (Yang et al., 2020; Lu et al., 2022). Interestingly, Y906 mutation in the wild-type CmTRPM8 (without the MHR1-3 region of XtTRPM8) is not sufficient to confer cold sensitivity to the resultant mutants (Lu et al., 2022). These results suggest that the modifications in sequence within the MHR1-3 region during evolution would be the first structural requirement that allows TRPM8 channels to be gated by cold. Meanwhile, the changes in the pore domain could serve to enhance the efficacy of cold activation to strengthen mammal TRPM8-dependent cold sensing (Lu et al., 2022).

## 5 Residues involved in voltage induced activation

TRPM8 is a weakly voltage-dependent channel that requires strong membrane depolarization to open (Brauchi et al., 2004; Voets et al., 2004). Which residues contribute to the voltage sensor has been the aim of different studies (Voets et al., 2007; Winking et al., 2012; Kalia and Swartz, 2013; Kühn et al., 2013). Voets and coworkers, guided by the similarities displayed between TRP channels and voltage-activated potassium channels, focused on the TRPM8 region corresponding to the voltage sensor in Kv1.2 channels. R842, H845, R851, K856, and R862, located within S4 and the S4-S5 linker in MmTRPM8 structures (Yin et al., 2022; Zhao et al., 2022), were identified as putative positive charges contributing to the voltage sensor. Among them, only the alanine substitution of R842 within the S4 and R856 in the S4-S5 linker resulted in ion channels with reduced gating charge (Voets et al., 2007). Unfortunately, the double mutation of R842A and R856A, which would help to assess their total contribution to the voltage sensor, generated a non-functional channel (Voets et al., 2007). However, considering an additive contribution of R842 and R856, they should be accountable only for 50%–70% of the gating charge, suggesting that other residues could also be responsible for voltage-induced TRPM8 activation (Voets et al., 2007). Furthermore, as discussed in previous sections, mutations of positive amino acids in the S4 and the S4-S5 linker strongly affect TRPM8 responses to cold and menthol, suggesting that these activators could act on TRPM8 through interaction with the voltage sensor (Voets et al., 2007).

However, positive amino acids within S4 do not contribute to a canonical voltage sensor unit as observed in classical voltage-dependent cation channels (Catterall, 2010). Charge reversal mutations of R842 (within S4) by aspartate or glutamate, retains wild-type TRPM8 voltage sensitivity if these mutations are compensated by the opposite charge reversal of D802 (D802R + R842D and D802R + R842E), suggesting that rather than positively charged residues in the S4 the combination of charged residues in S3 and S4 determines the voltage sensor function (Kühn et al., 2013). Moreover, introducing F839R, H845R, and T848K mutations into S4 to mimic the positive charge distribution observed in voltage-dependent potassium channels only caused a shift in the V_1/2_ toward more positive membrane potentials (Kühn et al., 2013).

## 6 Antagonists

Several TRPM8 inhibitors have been described in the last 20 years (reviewed by (Pérez De Vega et al., 2016; González-Muñiz et al., 2019; Izquierdo et al., 2021)). Based on the central structural scaffold, they have been classified into different groups (for a description of their structures and effects, see (Pérez de Vega et al., 2016)). Regarding the mechanism of action of these antagonists, an early work of Mälkiä and others showed that inhibitors such as BCTC and SKF96365 induce a rightward shift of the voltage activation curve of TRPM8, decreasing the probability of channel opening at physiological membrane potentials (Mälkiä et al., 2007). When cold or chemical agonists are co-applied with TRPM8 antagonists, their effects on the voltage activation curve are canceled, suggesting a shared molecular mechanism (Mälkiä et al., 2007). Part of this convergence could be explained by the fact that tyrosine 745, involved in menthol- and icilin-dependent gating, is also critical for the inhibition exerted by some antagonistic compounds, such as SKF96365 (Mälkiä et al., 2009). Nevertheless, inhibition by other antagonists, like BCTC, is not prevented by the mutation of this position (Mälkiä et al., 2009).

Diver and coworkers corroborated the idea that agonists and antagonists share the binding site. They evaluated the structure of the *Parus major* TRPM8 (PmTRPM8) complex with two structurally different antagonists: AMTB and TC-I 2014 (Table 1) (Diver et al., 2019). Like chemical TRPM8 activators, these two molecules fit near the membrane-cytosol interface within a pocket formed by residues of the VSLD and TRP domains (Diver et al., 2019; Yin et al., 2019). The authors proposed that the binding of the antagonists with the TRPM8 channel is facilitated through the complementarity shape displayed by this cavity rather than specific hydrogen bonds or ionic interactions (Diver et al., 2019). Interestingly, although these compounds are accommodated within the same pocket, their orientations differ (Diver et al., 2019), which could explain why the Y745H mutation could abrogate the inhibition exerted by some antagonists but not others. Since no significant alterations were observed between the ligand-free and the antagonist-bound structures, the authors suggest that possibly chemical antagonists, although structurally different, prevent TRPM8 gating by locking the channel in its apo state configuration (Diver et al., 2019).

## 7 TRPM8 desensitization mechanisms

One feature of TRPM8, reported since its initial characterization, is its Ca^2+^-dependent desensitization when activated by cold or menthol (McKemy et al., 2002; Chuang et al., 2004; Liu and Qin, 2005; Rohács et al., 2005; Mälkiä et al., 2007), which could result from the direct interaction of this ion with TRPM8 or Ca^2+^-dependent signaling cascades. The following section will discuss the molecular mechanisms proposed to explain this physiologically relevant form of regulation.

PI(_4,5_)P_2_ is a crucial functional regulator of TRPM8 activity (Liu and Qin, 2005; Rohács et al., 2005; Zakharian et al., 2010). Ca^2+^ influx through TRPM8 channels would activate the Ca^2+^-dependent phospholipase C (PLC), hydrolyzing PI(_4,5_)P_2_ into diacylglycerol (DAG) and inositol-1,4,5-triphosphate, inducing a decrease of PI(_4,5_)P_2_ levels at the plasma membrane and therefore a reduction in TRPM8 activity. In addition, this would also explain the rundown of the channel activity observed in excised patches, where lipids and proteins are dephosphorylated (Liu and Qin, 2005; Rohács et al., 2005). Several facts support this mechanism; for instance, PI(_4,5_)P_2_ depletion by poly-L-lysine increases the rundown of the channel (Liu and Qin, 2005; Rohács et al., 2005). Conversely, its activity is recovered after the application of PI(_4,5_)P_2_ (Liu and Qin, 2005; Rohács et al., 2005). In addition, the rundown was prevented by inhibiting PI(_4,5_)P_2_ dephosphorylation (Liu and Qin, 2005). Moreover, the reduction in PI(_4,5_)P_2_ levels using pharmacological tools that target the enzymes involved in this phospholipid metabolism or through the stimulation of receptor-mediated hydrolysis of PI(_4,5_)P_2_ downregulates TRPM8 function (Liu and Qin, 2005; Rohács et al., 2005; Daniels et al., 2009). In addition, no TRPM8 responses to menthol were observed after scavenging PI(_4,5_)P_2_; on the contrary, high concentrations (500 µM) of diC8 PI(_4,5_)P_2_ evoked TRPM8 currents even at temperatures above the temperature threshold of cells in control conditions, implying that PI(_4,5_)P_2_ is necessary for TRPM8 gating (Rohács et al., 2005). Finally, as in excised patches (Rohács et al., 2005), diC8 PI(_4,5_)P_2_ activates TRPM8 in lipid bilayers more effectively than other phosphoinositides, indicating a specific interaction of the channel with PI(_4,5_)P_2_ (Zakharian et al., 2010).

**FIGURE 3:**
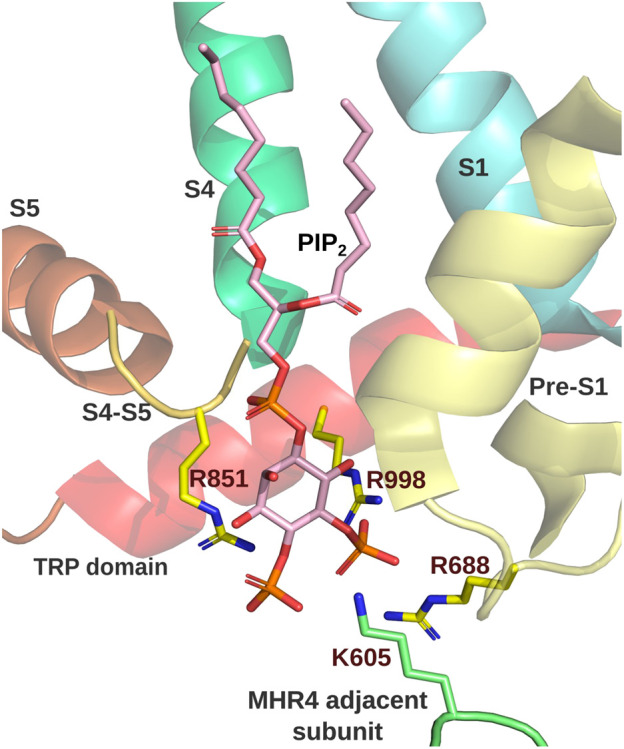
TRPM8 PI(_4,5_)P_2_ interacting site. PI_(4,5)_P_2_ binding site of MmTRPM8 (PDB ID: 8E4N). Residues interacting with PI_(4,5)_P_2_ are shown. R688 at the pre-S1 (yellow), R850, located at the junction between S4 (dark green) and S5 (orange), R998 within the TRP domain (red), and R605 from the MHR4 of the adjacent subunit (green) (Visualization in Pymol v2.5.4).

All these findings suggest that TRPM8 accommodates PI(_4,5_)P_2_ in its structure. From the beginning, positive residues located within the TRP domain attract the attention as putative interaction sites for PI(_4,5_)P_2_. Replacements of K995, R998, and R1008 by glutamine induced a right shift in their dose-response curve to diC8 PI(_4,5_)P_2_, suggesting decreased sensitivity to this molecule (Rohács et al., 2005). Although not all cryo-EM structure studies of TRPM8 channels captured the presence of PI(_4,5_)P_2_ (Diver et al., 2019; Zhao et al., 2022), some shed light on the actual binding site of PI(_4,5_)P_2_. The positively charged residues that interact with this molecule are R688 at the pre-S1, R850, located at the junction between S4 and S5, R997 (R998 in mouse ortholog) within the TRP domain, as suggested by (Rohács et al., 2005), and R605 from the MHR4 of the adjacent subunit (Figure 3). Consistently, K605Q, R850Q, and R997Q mutants exhibited a right-warded shift in the conductance-voltage curves, in agreement with defects in channel activation expected by the loss of the interaction between TRPM8 and PI(_4,5_)P_2_ (Yin et al., 2019).

However, a different mechanism to explain TRPM8 desensitization has been proposed. As mentioned below, several cryo-EM TRPM8 structures of avian and mammal TRPM8 agree with the existence of a Ca^2+^ coordination site involved in the icilin-dependent gating (Diver et al., 2019; Yin et al., 2019; Zhao et al., 2022), which could also be relevant for TRPM8 desensitization. As previously reported for TRPM2, TRPM4, and TRPM5 channels (Autzen et al., 2018; Huang et al., 2019; Ruan et al., 2021), a Ca^2+^ ion interacts with the cytosolic-facing cavity in the VSLD through the side chains of residues E782, Q785, N799, and D802 of FaTRPM8 (Yin et al., 2019) and MmTRPM8 (Zhao et al., 2022) (Figure 2B), that correspond to E773, Q776, N790, and D793 in the PmTRPM8 structure (Diver et al., 2019). In addition, two of these studies reported that Y793 (or Y784 in PmTRPM8) within the S2-S3 linker also contributes to the coordination sphere (Diver et al., 2019; Zhao et al., 2022). To assess the role of this Ca^2+^-binding site in Ca^2+^-dependent desensitization, Diver and collaborators mutated these five positions to alanine and estimated desensitization by measuring the currents at the beginning and the end of a 100 µM menthol application. Only Q776A, N790A, and Y793A significantly reduced desensitization compared to wild-type channels (Diver et al., 2019). Interestingly, in the same study, using a version of the parrot channel where an A796G mutation was introduced to make this ortholog icilin-sensitive, each of the five created mutants of the Ca^2+^-binding site prevented icilin-induced TRPM8 activation, including E773A and D793A (Diver et al., 2019). Since the last two mutants did not display significant alterations in desensitization, it prompted the authors to speculate that these two mechanisms could require high (activation by icilin) and low (desensitization) calcium binding affinities (Diver et al., 2019).

## 8 TRPM8 pore domain

**FIGURE 4:**
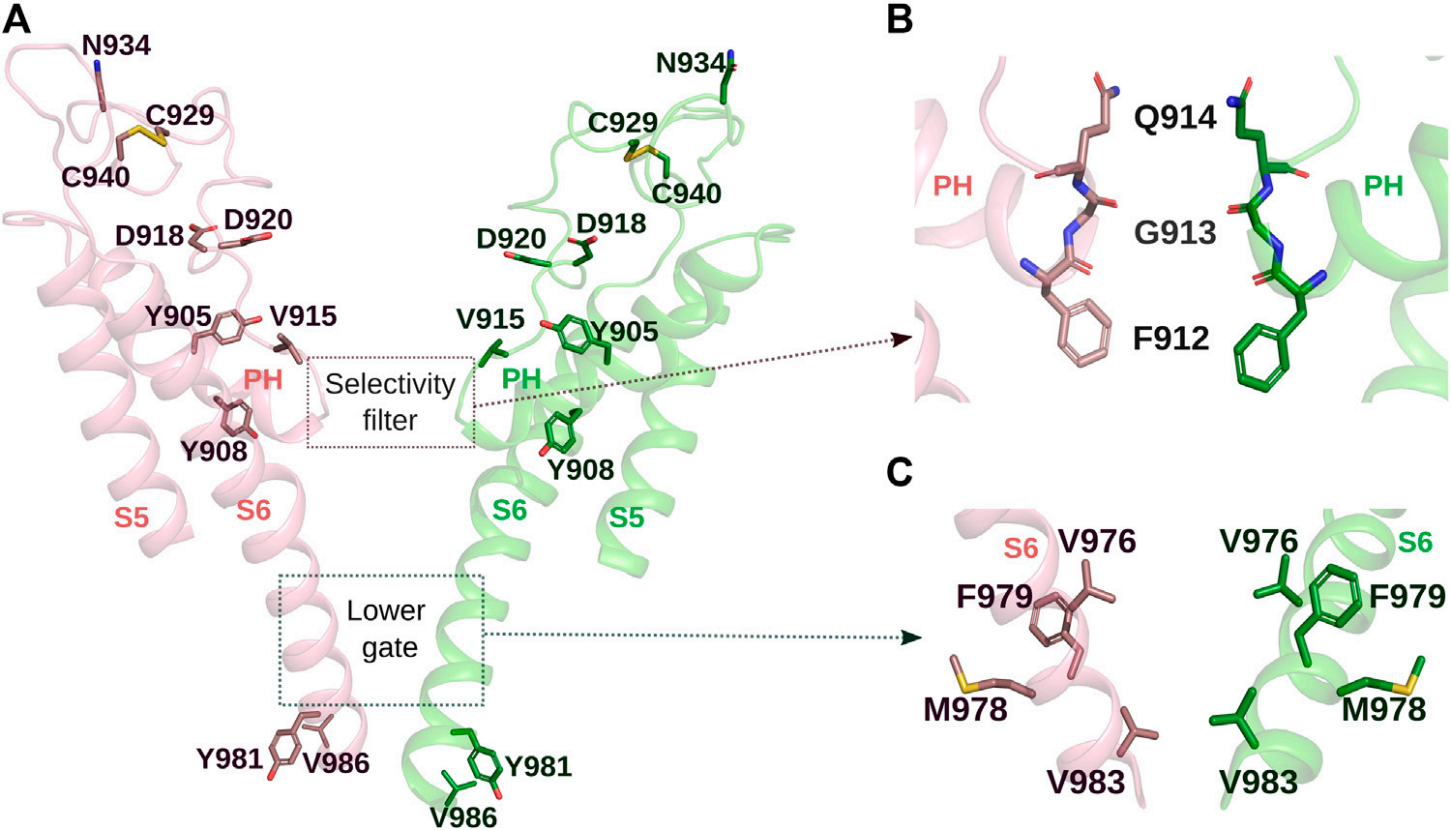
Pore domain of MmTRPM8. **(A)**. Representation of MmTRPM8’s ion conduction pathway with the front and rear subunits removed for clarity. Residues of mutants exhibiting major alterations in the responses of TRPM8 by cold or chemical agonists are shown as sticks (PDB ID: 7WRE). **(B)**. Close-up of the side chains of residues Q914, G913, and F912 in MmTRPM8 in the open state, which form the selectivity filter (PDB ID: 8E4L). **(C)**. Close-up view of the lower gate. Gate residues V976, M978, F979, and V983 are shown as sticks (PDB ID: 8E4L) (Visualization in Pymol v2.5.4).

The pore domain of TRPM8 is formed by S5 and S6, the interconnecting pore helix, and the outer pore, that show multiple negatively charged amino acids promoting the recruitment of cations to the pore (Diver et al., 2019; Zhao et al., 2022) (Figure 4A). In MmTRPM8 structures, two restrictions controlling the passage of ions are observed: the ^912^FGQ^914^ motif, similar to TRPM2 and TRPM4 channels (Guo et al., 2017; Yu et al., 2021), serves as a selectivity filter (Yin et al., 2022; Zhao et al., 2022) (Figure 4B), and the lower gate within the inner leaflet (Yin et al., 2022; Zhao et al., 2022) (Figure 4C).

Our understanding of how cold or chemical agonists lead TRPM8 channel opening has advanced thanks to the recent resolution of the open state. It has been proposed that chemical ligands binding at VSLD cytosolic-facing cavity may induce small local changes within this domain, which are transferred to the pore domain via the S4-S5 linker and the TRP domain, affecting TRPM8 channel gating (Xu et al., 2020; Yin et al., 2022; Zhao et al., 2022). In 2022, Yin and coworkers unveiled part of the conformational changes required for TRPM8 opening by chemical agonists, reporting three closed states (C_0_, C_1_, and C_2_) and one open conformation of MmTRPM8 observed after co-application of allyl isothiocyanate, that activates TRPM8 channel at millimolar concentrations (Janssens et al., 2016), cryosim-3, a novel TRPM8 agonist (Yang et al., 2017), and PI(_4,5_)P_2_ (Yin et al., 2022). In contrast to other TRP channels, the C_0_ conformation is characterized by a wide vestibule and a lower gate where M978 and F979 serve as gate residues (in PmTRPM8 M968 and F969) (Diver et al., 2019; Yin et al., 2022). C_1_ and C_2_ states observed during the binding of MmTRPM8 to PI(_4,5_)P_2_ and chemical agonists showed a rotation of the S6 helix, resulting in most of its hydrophobic residues facing the luminal part of the pore being replaced by negatively charged or polar amino acids, inducing a reduction in the pore cavity and the formation of the selectivity filter. This rotation causes a movement of M978, leading V983 and F979 to form a hydrophobic gate, different from the C_0_ state (Yin et al., 2022). Finally, another rotation is required to achieve the O state, where M978 and F979 change their position, moving away from the ion pathway (Yin et al., 2022). In contrast, V976 moves to the luminal part, setting a conduction point of ∼9.1 Å in diameter, which allows the passage of hydrated cations (Yin et al., 2022) (Figure 4C). In line with its participation in controlling the permeation pathway, V976 replacement by a lysine switched TRPM8 selectivity from cations to anions (Kühn et al., 2007). Interestingly, in Ca^2+^-bound structures, representing a desensitized state that resembled the open conformation, the V976 side chain (in PmTRPM8 V966) accounts for the only constriction along the ion conduction pore that reduced the radius to less than 1 Å (Diver et al., 2019). Moreover, during the gating of TRPM8, coils at the N- and C-termini of S6 in the C_0_ state become part of the S6, adding four helical turns to this transmembrane domain in the open configuration; therefore, E988 on the TRP domain in the C_0_ state became part of the C-terminus of S6 in the O state (Yin et al., 2022). Mutations of V976, M978, F979, and V983 to polar, negatively charged, and hydrophobic residues generated a broad range of phenotypes: from non-functional channels (F979A, F979D, and V983D), mutants displaying a right-shift in the voltage activation curve (V976D, V976T, V976F; M978D, M978F; F979T, F979L; V983A, V983T, V983L, and V983F), to even channels exhibiting increased basal currents at a negative potential (V976A) (Yin et al., 2022). Altogether, these results support the relevance of these residues as a structural determinant of TRPM8 gating. In that regard, a previous study also identified Y981 at the lower gate contributing to the gating energetics (Taberner et al., 2014). Interestingly, Y981E and Y981K rendered TRPM8 channels constitutively active (Taberner et al., 2014). In contrast, Y981F produced a no longer functional channel that reached the plasma membrane to the same extent as wild-type channels, suggesting that this residue determines TRPM8 gating (Taberner et al., 2014). Taberner and coworkers also showed that V986L mutation induced a rightward shift in the voltage activation curve (Taberner et al., 2014). The TRPM8 homology model used in this study indicated that V986 interacted with the TRP domain and the S4–S5 linker and predicted that the leucine side chain would not fit properly in the cavity where V986 is accommodated, explaining the effects of V986L mutation in TRPM8 gating (Taberner et al., 2014). Notably, the MmTRPM8 cryo-EM structures in the close and open states corroborated that C-terminal residues of S6 are pivotal for TRPM8 activation through their interactions with the S4-S5 linker and TRP domain (Yin et al., 2022).

In addition, changes in several amino acids within the outer pore domain cause a different impact on channel function. Neutralizing D918 and D920, positioned adjacent to the selectivity filter (Figure 4A), strongly reduced TRPM8 responses to cold, menthol, or icilin (Bidaux et al., 2015). Interestingly, replacing D918 with alanine, asparagine, or glutamate did not cause a significant alteration. In contrast, D920A mutant displayed a reduction in the responses that was increased in the double mutant D918A + D920A, and the D920 substitution by asparagine almost abolished TRPM8 function (Bidaux et al., 2015). In the same study, the effect of mutating position Y905 and Y908 within the pore helix were explored (Figure 4A). While the Y905A mutation generates a non-functional channel, Y908A substitution results in an insensitive channel to cold and menthol that exhibited a robust response to icilin, suggesting that this tyrosine has an essential role in the gating of TRPM8 by cold and menthol (Bidaux et al., 2015).

Importantly, amino acids of the outer pore have post-translational modifications. N-glycosylation of TRPM8 occurs at N934, in the third extracellular loop (Dragoni et al., 2006; Erler et al., 2006) (Figure 4A), in both recombinant and native membranes (Dragoni et al., 2006; Erler et al., 2006; Pertusa et al., 2012). Protein extracts from recombinant and native systems usually display two or three distinguishable bands in a Western blot analysis, corresponding to different maturation stages of the TRPM8 biogenesis. The lowest band corresponds to the non-glycosylated channel, the intermediate band results from the cotranslational transfer of a mannose-rich precursor to the TRPM8 during synthesis in the endoplasmic reticulum (ER) compartment, and the higher band represents the more mature N-glycosylated form, containing terminal sialic acid residues, generated during TRPM8 transit through the Golgi complex (Morenilla-Palao et al., 2009; Pertusa et al., 2012). Importantly the unglycosylated TRPM8 mutant (N934Q) exhibits smaller responses to agonists (Erler et al., 2006; Pertusa et al., 2012). This reduction in the responses, observed in recombinant and native systems, could be explained by a direct effect of N-glycosylation on TRPM8 biophysical properties since lack of N-glycosylation shifts V_1/2_ almost 60 mV toward more positive membrane potentials (Pertusa et al., 2012). Finally, flanking the TRPM8 N-glycosylation, C929 and C940 are linked by a disulfide bond, creating a loop with a complex N-glycosylation to its apex (Dragoni et al., 2006) (Figure 4A), a finding corroborated by Cryo-EM studies (Diver et al., 2019; Zhao et al., 2022). The formation of this bridge is essential to this ion channel function. In two studies where these two cysteines were mutated, this manipulation generated non-functional channels, although the trafficking of the mutant channels to the plasma membrane was not prevented (Dragoni et al., 2006; Bidaux et al., 2015).

## 9 Discussion

Identifying amino acids and regions involved in the function and gating of TRPM8 channels is the first step in the search for new compounds targeting this ion channel. This review summarized TRPM8 residues and domains contributing to its responses to chemical and physical stimuli and those related to the desensitization process in light of the recent cryo-EM structures of TRPM8 channels.

One limitation of these studies is that validating the specific role of different amino acids in TRPM8 activity usually relies on the functional characterization of mutants. Although single-point mutagenesis and the construction of chimeric channels have been critical to unraveling regions or amino acids related to the gating of TRP channels and other ion channels, these strategies have not always been successful in TRPM8. When examining the literature on TRPM8, it is not uncommon to find reports of mutations resulting in non-functional channels (see Supplementary Table S1 summarizing some of them). This ion channel is relatively prone to yield a non-functional phenotype after different manipulations, from single-point mutagenesis to deletions or substitutions of a few amino acids in its cytosolic or transmembrane domains. In most cases, the loss of function classification came from the absence of currents of these mutants in patch-clamp experiments, which could result from defective trafficking to the plasma membrane, impaired function, or both. Although interpreting data from mutagenesis experiments requires caution, drawing conclusions from the failure to record discernible currents is substantially more difficult. Mutations altering the normal trafficking aside, non-active channels could be categorized into two main groups: in the first group, the mutation only causes defects in the activation of TRPM8, not disturbing its normal biogenesis and trafficking to the cell surface. In the second group, the changes in the protein sequence of TRPM8 compromised tetramerization or generated misfolding, often resulting in their accumulation in the endoplasmic reticulum (ER). Attending only to the loss of the functional phenotype challenges the discrimination if the mutation impacts the quaternary structure that impairs function and induces ER retention, interferes with some steps of the proper biogenesis, or only affects its function but not its trafficking. The latter case could be more informative, since at least a significant impact of the mutation in the overall structure is excluded. Albeit indirect, an easy way to distinguish between these situations is by assessing the glycosylation state (Pertusa et al., 2014). If the mature-glycosylated band is absent in Western blot analyses, it suggests that the channels are retained in the ER compartment (Pertusa et al., 2014). In recombinant systems, functional TRPM8 activity from the ER can be detected by Ca^2+^-imaging when cold and menthol stimuli are applied simultaneously (Pertusa et al., 2012). If the mutation affects the proper folding and assembly, compromising TRPM8 function, no responses will be observed when a combination of both stimuli is applied.

As discussed below, constructing chimeras using orthologs reduced the chances of obtaining non-functional channels when long regions of TRPM8 must be substituted. Although this review mainly focused on using chimeras to identify regions related to TRPM8 thermal response, orthologs also exhibited differences regarding their chemical sensitivity. As mentioned, unlike mammalian TRPM8, avian TRPM8 did not respond to icilin (Chuang et al., 2004; Selescu et al., 2013; Diver et al., 2019; Yin et al., 2019). In addition, concentration-response curves show that GgTRPM8 is more sensitive to menthol than murine TRPM8 channels (Chuang et al., 2004; Myers et al., 2009; Yamamoto et al., 2016; Pertusa et al., 2018), probably due to differences in residues within the VLSD (Pertusa et al., 2018). These discrepancies among orthologs could account for some of the differences in the impact of a given mutation upon the activation by chemical agonists observed in Table 3. Moreover, comparing MmTRPM8, FaTRPM8, and PmTRPM8 cryo-EM structures also showed differences (Yin et al., 2018; 2019; 2022; Diver et al., 2019; Zhao et al., 2022). For instance, MmTRPM8 structures obtained in the absence of ligands revealed a 3_10_ helical conformation in the C-terminus of S4 and the presence of the typical S4-S5 linker seen in TRP channels that results from the S5 bending (Yin et al., 2022; Zhao et al., 2022). In contrast, FaTRPM8 and PmTRPM8 in a free ligand state showed a straight S5 and an α-helical C-terminus of S4. In avian TRPM8 channels, the S5 bent and the S4 3_10_ helical structure only were observed in the icilin- PI(_4,5_)P_2_ –Ca^2+^ complex (PDB ID: 6NR3) or in a Ca^2+^-bound desensitized state (PDB ID: 6O77**)** (Diver et al., 2019; Yin et al., 2019). Since in one of the MmTRPM8 ligand free structures (PDB ID: 8E4O) a putative PI(_4,5_)P_2_ molecule was resolved, although no additional PI(_4,5_)P_2_ was included in the sample, this conformation was attributed to the interaction of TRPM8 to endogenous PI(_4,5_)P_2_ (Yin et al., 2022). The authors proposed that MmTRPM8 shows higher affinity to PI(_4,5_)P_2_ than FaTRPM8, which in turn could influence the role of this phosphoinositide as a regulator of this ion channel activity (Yin et al., 2022), explaining, at least in part, some of the functional differences exhibited by these orthologs (Yin et al., 2022). However, it has to be noted that no PI(_4,5_)P_2_ molecule was unambiguously identified in the structures from Zhao’s study, where the canonical S4-S5 linker and the S4 3_10_ helical conformation were also observed (Zhao et al., 2022). Nevertheless, *in silico* approaches to finding novel TRPM8 modulators must consider the structural differences among orthologs. Although the HsTRPM8 structure has not been resolved, the high homology among the TRPM8 sequence from *Ficedula albicollis*, *Parus major*, and *Mus musculus* allows the generation of an accurate homology model for the human TRPM8 channel to undertake this task, as it has been shown in (Beccari et al., 2017; Blair Journigan et al., 2020; Talarico et al., 2020).

Why is it important to explore new therapeutic strategies for pathologies where TRPM8 is involved? Although the number of studies showing the potential of TRPM8 as a relevant target to treat specific diseases has increased in the last years, the clinical use of known TRPM8 agonists and antagonists has faced several drawbacks (for a comprehensive review, see (Izquierdo et al., 2021)). Some pathologies related to the functional upregulation of TRPM8 function, like ocular dysesthesias, or painful cold hypersensitivity (Knowlton et al., 2010; Su et al., 2011; Piña et al., 2019), would require the administration of TRPM8 antagonists. Meanwhile, an agonist could be recommended in pathologic scenarios that originate from the negative regulation of TRPM8 activity, such as some forms of dry eye disease (Belmonte et al., 2017). In both cases, the modulatory effect on the channel’s physiological activity must be considered. This could explain why topical administration of drugs targeting TRPM8 channels, which only reach the nerve endings of primary sensory neurons, alleviates some types of pain. In contrast, systemic treatments which could affect TRPM8 activity in the central nervous system or other tissues where this channel is expressed produce important secondary effects (Izquierdo et al., 2021). Moreover, some common TRPM8 modulators are unspecific since they activate or inhibit other ion channels, contributing to the side effects observed in clinical trials and preventing their progression into clinical use (Izquierdo et al., 2021). In some cases, administering a modulator *in vivo* requires such high concentrations for the unbound plasma concentrations to be effective that it becomes toxic (Izquierdo et al., 2021).

Therefore, there is still room for preclinical development of new TRPM8 modulators that could pass clinical trials. The information recapitulated here could be helpful to assist the design of structural-based chemical modifications of known TRPM8 agonists and antagonists to improve drug potency, specificity, stability, solubility, focal availability, or the *in silico* high throughput screening for novel and rationally designed modulators of TRPM8 function.
