# Supplementary material for: Molecular determinants of TRPM8 function: key clues for a cool modulation
Source: Front Pharmacol. 2023 Jun 14;14:1213337. doi: 10.3389/fphar.2023.1213337 (PMC10301734; doi:10.3389/fphar.2023.1213337)
Supplement: Supplementary file 1 [file DataSheet1.PDF]

Supplementary Material

Molecular determinants of TRPM8 function: key clues for a cool modulation.

María Pertusa[1,2,3,*], Jocelyn Solorza[2,4], Rodolfo Madrid[1,2,3]

*** Correspondence:**
María Pertusa
maria.pertusa@usach.cl

1 Supplementary Table 1. Mutations, deletions, and chimeras exhibiting non-functional phenotype

Construct/ Mutant/ Chimera	Domain	Ortholog	Functional evaluation	Plasma membrane expression / Glycosylation state	Reference
**Δ41-48**	N-terminus	MmTRPM8	Ca^{2+}-imaging	Immature glycosylation	(Pertusa et al., 2014)
**Δ41-57**	N-terminus	MmTRPM8	Ca^{2+}-imaging	Immature glycosylation	(Pertusa et al., 2014)
**Δ49-57**	N-terminus	MmTRPM8	Ca^{2+}-imaging	Immature glycosylation	(Pertusa et al., 2014)
**Δ 1-86**	N-terminus	RnTRPM8	Patch-clamp recording	No plasma membrane expression	(Phelps and Gaudet, 2007)
**Δ1-116**	N-terminus	RnTRPM8	Patch-clamp recording	No plasma membrane expression	(Phelps and Gaudet, 2007)
**Δ1-245**	N-terminus	RnTRPM8	Patch-clamp recording	No plasma membrane expression	(Phelps and Gaudet, 2007)
**Δ1-345**	N-terminus	RnTRPM8	Patch-clamp recording	No plasma membrane expression	(Phelps and Gaudet, 2007)
**W682A**	N-terminus	RnTRPM8	Ca^{2+}-imaging<br>Patch-clamp recording	Plasma membrane expression	(Zheng et al., 2018)

**D835R**	S4	HsTRPM8	Not reported	Immature glycosylation	(Kühn et al., 2013)
**D802R+ D835R**	S3/S4	HsTRPM8	Not reported	Immature glycosylation	(Kühn et al., 2013)
**R842D**	S4	HsTRPM8	Ca^{2+}-imaging Patch-clamp recording	Immature glycosylation	(Kühn et al., 2013)
**R842E**	S4	HsTRPM8	Ca^{2+}-imaging Patch-clamp recording	Immature glycosylation	(Kühn et al., 2013)
**R842A+ K856A**	S4/ S4-S5 linker	HsTRPM8	Patch-clamp recording	Not reported	(Voets et al., 2007)
**R851A**	S4-S5 linker	HsTRPM8	Patch-clamp recording	Not reported	(Voets et al., 2007)
**Y905A**	Pore-helix	HsTRPM8	Patch-clamp recording	Plasma membrane expression	(Bidaux et al., 2015)
**C929A**	Outer pore	MmTRPM8	Ca^{2+}-imaging	Plasma membrane expression	(Dragoni et al., 2006)
**C940A**	Outer pore	MmTRPM8	Ca^{2+}-imaging	Plasma membrane expression	(Dragoni et al., 2006)
**C929A+ C940A**	Outer pore	MmTRPM8	Ca^{2+}-imaging	Plasma membrane expression	(Dragoni et al., 2006)
**C940R**	Outer pore	HsTRPM8	Patch-clamp recording	Plasma membrane expression	(Bidaux et al., 2015)
**C940G**	Outer pore	HsTRPM8	Patch-clamp recording	Plasma membrane expression	(Bidaux et al., 2015)
**R950E**	Outer pore	HsTRPM8	Patch-clamp recording	Plasma membrane expression	(Bidaux et al., 2015)

**N934Q+ H946N**	Outer pore	HsTRPM8	Patch-clamp recording	No Plasma membrane expression	(Erler et al., 2006)
**F979A**	S6	MmTRPM8	Patch-clamp recording	Not reported	(Yin et al., 2022)
**F979D**	S6	MmTRPM8	Patch-clamp recording	Not reported	(Yin et al., 2022)
**Y981F**	S6	RnTRPM8	Patch-clamp recording	Plasma membrane expression	(Taberner et al., 2014)
**V983D**	S6	MmTRPM8	Patch-clamp recording	Not reported	(Yin et al., 2022)
**V986A**	S6	RnTRPM8	Patch-clamp recording	Not reported	(Taberner et al., 2014)
**V986G**	S6	RnTRPM8	Patch-clamp recording	Not reported	(Taberner et al., 2014)
**V986F**	S6	RnTRPM8	Patch-clamp recording	Not reported	(Taberner et al., 2014)
**R998A**	TRP domain	RnTRPM8	Ca^{2+}-imaging Patch-clamp recording	Plasma membrane expression	(Zheng et al., 2018)
**∆992-1104**	C-terminus	RnTRPM8	Patch-clamp recording	Plasma membrane expression	(Phelps and Gaudet, 2007)
**∆1070-1104**	C-terminus	RnTRPM8	Patch-clamp recording	Plasma membrane expression	(Phelps and Gaudet, 2007)
**∆1088-1104**	C-terminus	RnTRPM8	Patch-clamp recording	Not reported	(Phelps and Gaudet, 2007)
**K1027Q**	C-terminus	RnTRPM8	Patch-clamp recording	Not reported	(Rohács et al., 2005)
**L1089P**	C-terminus	HsTRPM8	Patch-clamp recording	No mature-glycosylation	(Erler et al., 2006)

				Plasma membrane expression	
**TRPM2-TRPM8 chimeras**		MmTRPM8	Not reported	Not reported	(Bandell et al., 2006)
**TRPM2-TRPM8 chimeras**	N-terminus	MmTRPM8	Ca^{2+}-imaging	Immature glycosylation	(Pertusa et al., 2014)
**TRPM2-TRPM8 chimeras**	Pore	HsTRPM8	Patch-clamp recording	Not reported	(Kühn et al., 2007)
**TRPM5-TRPM8 chimeras**	N-terminus/ C-terminus	HsTRPM8	Not reported	Not reported	(Voets et al., 2007)
**TRPM8-S1-S4 TRPM5**	VSLD	HsTRPM8	Patch-clamp recording	No plasma membrane expression	(Voets et al., 2007)
**TRPM8-S5-S6 TRPM5**	S5-S6	HsTRPM8	Patch-clamp recording	Plasma membrane expression	(Voets et al., 2007)
**TRPM8-S4 TRPM5**	S4	HsTRPM8	Patch-clamp recording	Plasma membrane expression	(Voets et al., 2007)
**TRPM8-S4a TRPM5**	S4	HsTRPM8	Patch-clamp recording	Plasma membrane expression	(Voets et al., 2007)
**TRPM8-S4c TRPM5**	S4	HsTRPM8	Patch-clamp recording	Plasma membrane expression	(Voets et al., 2007)
**Kv2.1-TRPM8**	S1-S6	RnTRPM8	Patch-clamp recording	Not reported	(Kalia and Swartz, 2013)

References

Bandell M, Dubin AE, Petrus MJ, Orth A, Mathur J, Hwang SW, Patapoutian A (2006) High-throughput random mutagenesis screen reveals TRPM8 residues specifically required for activation by menthol. Nat Neurosci 9:493–500.

Bidaux G, Sgobba M, Lemonnier L, Borowiec A-S, Noyer L, Jovanovic S, Zholos A V., Haider S (2015) Functional and Modeling Studies of the Transmembrane Region of the TRPM8 Channel. Biophys J 109:1840–1851.

Dragoni I, Guida E, McIntyre P (2006) The cold and menthol receptor TRPM8 contains a functionally important double cysteine motif. J Biol Chem 281:37353–37360.

Erler I, Al-Ansary DMM, Wissenbach U, Wagner TFJ, Flockerzi V, Niemeyer BA (2006) Trafficking and assembly of the cold-sensitive TRPM8 channel. J Biol Chem 281:38396–38404.

Kalia J, Swartz KJ (2013) Exploring structure-function relationships between TRP and Kv channels. Sci Reports 2013 31 3:1–9.

Kühn FJP, Knop G, Lückhoff A (2007) The transmembrane segment S6 determines cation versus anion selectivity of TRPM2 and TRPM8. J Biol Chem 282:27598–27609.

Kühn FJP, Winking M, Kühn C, Hoffmann DC, Lückhoff A (2013) Surface expression and channel function of TRPM8 are cooperatively controlled by transmembrane segments S3 and S4. Pflugers Arch Eur J Physiol 465:1599–1610.

Pertusa M, González A, Hardy P, Madrid R, Viana F (2014) Bidirectional Modulation of Thermal and Chemical Sensitivity of TRPM8 Channels by the Initial Region of the N-terminal Domain. J Biol Chem 289:21828.

Phelps CB, Gaudet R (2007) The role of the N terminus and transmembrane domain of TRPM8 in channel localization and tetramerization. J Biol Chem 282:36474–36480.

Rohács T, Lopes CMB, Michailidis I, Logothetis DE (2005) PI(4,5)P2 regulates the activation and desensitization of TRPM8 channels through the TRP domain. Nat Neurosci 2005 85 8:626–634.

Taberner FJ, López-Córdoba A, Fernández-Ballester G, Korchev Y, Ferrer-Montiel A (2014) The Region Adjacent to the C-end of the Inner Gate in Transient Receptor Potential Melastatin 8 (TRPM8) Channels Plays a Central Role in Allosteric Channel Activation. J Biol Chem 289:28579–28594.

Voets T, Owsianik G, Janssens A, Talavera K, Nilius B (2007) TRPM8 voltage sensor mutants reveal a mechanism for integrating thermal and chemical stimuli. Nat Chem Biol 2007 33 3:174–182.

Yin Y, Zhang F, Feng S, Butay KJ, Borgnia MJ, Im W, Lee S-Y (2022) Activation mechanism of the mouse cold-sensing TRPM8 channel by cooling agonist and PIP2. Science (80-) 378.

Zheng W, Cai R, Hofmann L, Nesin V, Hu Q, Long W, Fatehi M, Liu X, Hussein S, Kong T, Li J, Light PE, Tang J, Flockerzi V, Tsiokas L, Chen XZ (2018) Direct Binding between Pre-S1 and TRP-like Domains in TRPP Channels Mediates Gating and Functional Regulation by PIP2. Cell Rep 22:1560–1573.
